# The dual effects of anesthetics on glial cells: a review of neuroprotection and neurotoxicity

**DOI:** 10.3389/fphar.2026.1724695

**Published:** 2026-01-26

**Authors:** Xiaodong Wang, Minghe Zhao, Zhihui Liu

**Affiliations:** 1 Affiliated Baotou Clinical College of Inner Mongolia Medical University, Baotou, China; 2 Department of Anesthesiology, Baotou Central Hospital, Baotou, China

**Keywords:** anesthetics, astrocytes, central nervous system, glial cells, microglia, neuroinflammation, neurotoxicity, oligodendrocytes

## Abstract

Glial cells—comprising astrocytes, microglia, and oligodendrocytes—are fundamental to central nervous system (CNS) homeostasis, yet their interactions with anesthetics are not fully elucidated. This review narratively synthesizes current evidence on the differential effects of local and general anesthetics on these cells, revealing a complex duality of neuroprotective and neurotoxic outcomes. Local anesthetics such as lidocaine can confer protection by inducing astrocytic autophagy and suppressing microglial pro-inflammatory responses, whereas bupivacaine may impair astrocytic mitochondrial function and potentiate excitotoxicity. Conversely, general anesthetics exhibit divergent impacts: propofol demonstrates protective properties against oxidative stress and neuroinflammation, but isoflurane often induces astrocytic cytotoxicity, activates microglia via the NF-κB pathway, and triggers apoptosis in developing oligodendrocytes, thereby disrupting myelination. These effects are critically influenced by anesthetic type, concentration, exposure duration, and the pathological context. Our analysis underscores the necessity of understanding these glial-centric mechanisms to optimize anesthetic safety, particularly for vulnerable populations such as the young and the elderly. Ultimately, advancing the knowledge of how anesthetics modulate glial cell function is pivotal for developing personalized anesthesia strategies that minimize neurotoxicity and harness potential protective effects, thereby improving postoperative neurological outcomes and guiding future translational research.

## 1 Introduction

### 1.1 Background on glial cells

Glial cells—including astrocytes, microglia, and oligodendrocytes—constitute essential non-neuronal components of the central nervous system (CNS). Beyond their classical roles in structural support and insulation, glial cells actively maintain CNS homeostasis by regulating extracellular ion balance, neurotransmitter recycling, and blood–brain barrier integrity (Peng et al., 2023; Liu et al., 2023; Kwon and Koh, 2020). Astrocytes modulate synaptic transmission and neurovascular coupling; microglia serve as resident immune cells, surveilling the microenvironment and responding to injury; and oligodendrocytes myelinate axons to ensure efficient signal conduction. Emerging evidence highlights glial cells as dynamic regulators of neuroinflammation, neuroprotection, and neural plasticity, with their dysfunction implicated in a spectrum of CNS disorders, from neurodegenerative diseases to chronic pain (Donnelly et al., 2020; Sanmarco et al., 2021; Uddin and Lim, 2022). Moreover, bidirectional glia–neuron signaling is increasingly recognized as critical for synaptic function and network stability (Guttenplan and Liddelow, 2019; Maysinger and Ji, 2019). Thus, elucidating glial biology is pivotal for understanding both CNS physiology and pathology.

### 1.2 Overview of anesthetics

Anesthetics, indispensable in clinical practice, are broadly categorized into local and general agents based on their site and mechanism of action. Local anesthetics such as lidocaine and bupivacaine primarily block voltage-gated sodium channels on neuronal membranes, preventing action potential propagation and producing reversible regional analgesia (Macfarlane et al., 2021; Wadlund, 2017). In contrast, general anesthetics (e.g., propofol, isoflurane, sevoflurane) induce a reversible state of unconsciousness and analgesia by modulating key neurotransmitter receptors, including GABA_a_ and NMDA receptors (Hemmings et al., 2019; Mashour, 2024).

Traditionally, research on anesthetic mechanisms has focused on neuronal targets. However, growing data indicate that anesthetics also exert direct and indirect effects on glial cells, with functional consequences for CNS homeostasis. For instance, general anesthetics alter astrocytic calcium dynamics and microglial activity, thereby influencing neuroinflammatory pathways and synaptic plasticity (Brécier et al., 2023; Lim et al., 2021). Local anesthetics may affect glial function through sodium channel modulation and disruption of glia–neuronal communication (Berde, 2015; Fallon et al., 2024). These observations underscore the need to systematically examine how different anesthetic classes impact glial physiology and contribute to both neuroprotective and neurotoxic outcomes.

### 1.3 Relevance of studying anesthetic effects on glial cells

The distinct effects of local and general anesthetics on glial cell function carry significant implications, given the essential contributions of glial cells to CNS homeostasis, neuroinflammation, neuroprotection, and repair. Since these cells are further implicated in a range of CNS pathologies—such as neurodegenerative disorders and brain injury—understanding how anesthetics regulate glial activity may help reduce adverse effects and refine clinical anesthetic strategies for better patient outcomes (Bossuyt et al., 2023; Deng et al., 2024).

Recent studies reveal distinct modulation of glial cell signaling, calcium dynamics, and inflammatory responses by local versus general anesthetics, underscoring the necessity of mechanistic investigations to clarify these cellular interactions (Keshavarz, 2017; Koizumi and Hirayama, 2022). Elucidating these mechanisms will inform the design of safer and more effective anesthetic strategies that account for the specialized functions of glial cells, thereby improving patient care and advancing anesthetic practice.

**FIGURE 1:**
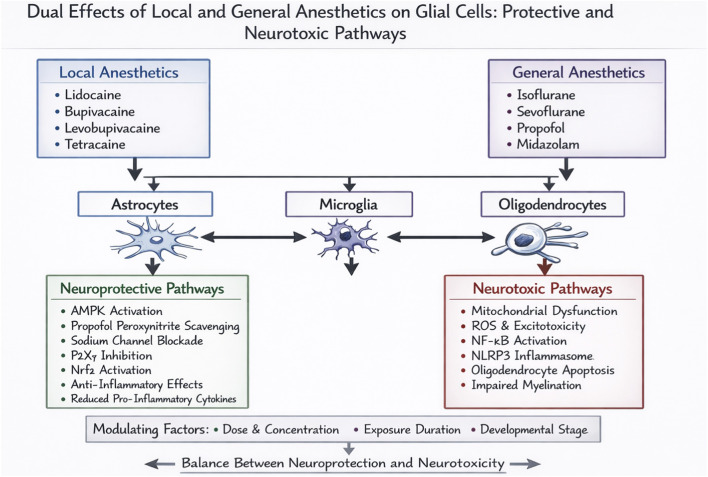
Dual Effects of Local and General Anesthetics on Glial Cells: Protective and Neurotoxic Pathways. This graphical summary illustrates the dual pathways through which anesthetic agents may exert effects on the central nervous system. At the top, the pharmacological sources are categorized into Local Anesthetics (e.g., Lidocaine, Bupivacaine) and General Anesthetics (e.g., Isoflurane, Propofol). These agents primarily target three central glial cell types: Astrocytes, Microglia, and Oligodendrocytes. Glial activation can subsequently initiate two opposing sets of pathophysiological pathways.

Despite accumulating evidence, a systematic analysis of the dual—often opposing—neuroprotective and neurotoxic effects of anesthetics on glial cells is lacking. This duality is highly context-dependent, hinging on specific agents, concentrations, and pathological states. To address this gap, this review synthesizes current evidence by comparing anesthetic actions on each major glial cell type—astrocytes, microglia, and oligodendrocytes. Crucially, within each cell type, we organize the discussion around key mechanistic themes (e.g., modulation of autophagy, mitochondrial function, inflammatory signaling, and apoptosis) that underlie the divergent outcomes elicited by different local (Section 2) and general (Section 3) anesthetics. This thematic approach aims to clarify the conditions that favor protection versus toxicity, identify knowledge gaps, and inform the development of safer anesthetic strategies (Figure 1).

## 2 Local anesthetics and glial cell function

### 2.1 Effects on astrocytes

Local anesthetics elicit dual effects on astrocyte function, ranging from protective to detrimental. These divergent outcomes can be traced to actions on two broad mechanistic fronts: the promotion of cytoprotective processes and the disruption of metabolic and ionic homeostasis. The specific agent and its concentration critically determine the balance.

Lidocaine, a widely used local anesthetic, has been extensively investigated for its effects on astrocytes. Studies demonstrate that lidocaine can induce astrocytic autophagy, a process shown to exert neuroprotective effects in models of chronic constriction injury (CCI)-induced neuropathic pain (Yuan and Fei, 2021). Autophagy, an essential cellular degradation pathway for removing damaged organelles and proteins, can be activated by lidocaine. This induction suggests a mechanism through which the anesthetic may alleviate neuropathic pain by maintaining astrocyte viability. Furthermore, lidocaine suppresses pro-inflammatory cytokine production in astrocytes, an effect particularly relevant in neuroinflammatory contexts such as chronic pain and neurodegenerative disorders (Zhang et al., 2017; Zheng et al., 2019). This anti-inflammatory action involves the suppression of key signaling pathways—including AMP-activated protein kinase (AMPK) and suppressor of cytokine signaling 3 (SOCS3)—that regulate inflammatory responses in astrocytes (Zhang et al., 2017).

In contrast, local anesthetics such asIn contrast to the cytoprotective and anti-inflammatory profile of lidocaine, other local anesthetics can perturb astrocytic homeostasis, predisposing to neurotoxicity. Bupivacaine exerts more complex influences on astrocyte function. At low concentrations, bupivacaine ameliorates conditions such as painful diabetic neuropathy through modulation of microglial signaling pathways. However, at higher concentrations, it can indirectly affect astrocytes and produce potentially detrimental effects. For example, bupivacaine impairs astrocytic mitochondrial function, which may subsequently enhance glutamate-evoked intracellular calcium signaling in adjacent neurons (Xing et al., 2018). Such perturbation of calcium homeostasis promotes excitotoxicity—a process in which excessive neuronal activation by glutamate causes cellular damage or death. Bupivacaine impairs mitochondrial function by uncoupling oxidative phosphorylation and promoting reactive oxygen species (ROS) production (Lai et al., 2022). Mitochondrial dysfunction reduces ATP synthesis, compromising astrocytic ability to maintain ion gradients. This leads to impaired glutamate uptake via EAATs (Beard et al., 2021), resulting in extracellular glutamate accumulation. Concurrently, disrupted calcium buffering in astrocytes enhances neuronal calcium influx through NMDA receptors, precipitating excitotoxicity. These events illustrate how anesthetic-induced astrocytic metabolic failure can propagate neuronal injury. Furthermore, prolonged bupivacaine exposure reduces glycine transporter 1 (GlyT1) expression in astrocytes, potentially exacerbating excitotoxicity through impaired glycine reuptake and disrupted regulation (Werdehausen et al., 2012). These findings demonstrate the dual nature of local anesthetics in astrocyte regulation: they may offer therapeutic benefits at low concentrations yet produce adverse effects at elevated doses or with prolonged use.

The differential effects of local anesthetics on astrocytes underscore the need for precise control of dosage and exposure duration in clinical practice. Although these agents can provide neuroprotection through astrocyte modulation, their potential to provoke excitotoxicity and other forms of cellular impairment requires careful management—particularly in vulnerable patients, such as those with pre-existing neurodegenerative disorders.

The differential effects of lidocaine and bupivacaine on astrocytes highlight a critical principle in anesthetic-glial interactions: concentration and context determine functional outcomes. While lidocaine promotes autophagy and suppresses inflammation—potentially useful in neuropathic pain—bupivacaine at high concentrations disrupts mitochondrial integrity and calcium homeostasis, favoring excitotoxicity. This contrast underscores that not all local anesthetics are interchangeable in their glial-modulatory profiles. Furthermore, the opposing actions on astrocytic glutamate and glycine regulation suggest that anesthetic selection should consider pre-existing neuronal vulnerability, such as in neurodegenerative conditions where excitatory imbalance is already present.

### 2.2 Effects on microglia

Local anesthetics modulate microglial activity in a context-dependent manner. Their effects largely diverge into two categories: suppression of neuroinflammatory activation and, less commonly, induction of direct cytotoxicity. With outcomes ranging from beneficial to detrimental based on the specific agent and physiological conditions.

Lidocaine suppresses microglial activation and reduces pro-inflammatory cytokine production in multiple neuropathic pain models. For example, pretreatment with lidocaine decreases spinal microglial activation and alleviates associated pain-related behavioral responses (Cheng et al., 2011). This effect appears to involve modulation of sodium channel expression and inhibition of key signaling pathways—particularly p38 MAPK—that drive microglial activation (Gu et al., 2008). Lidocaine attenuates microglial activation through voltage-gated sodium channel blockade, which reduces Na^+^ influx and subsequent downstream signaling (Ma et al., 2021). This inhibition decreases phosphorylation of p38 MAPK, a key regulator of inflammatory cytokine transcription. Suppression of p38 MAPK leads to reduced nuclear translocation of NF-κB and diminished expression of TNF-α and IL-1β (Kuo et al., 2023). Concurrently, lidocaine upregulates SOCS3, which further inhibits JAK/STAT signaling and cytokine release. Together, these pathways synergistically limit neuroinflammatory amplification, providing a mechanistic basis for its utility in neuropathic pain models. Lidocaine also suppresses microglial release of pro-inflammatory cytokines, including TNF-α and IL-1β, thereby potentially contributing to its neuroprotective role during neuroinflammation (Jeong et al., 2012; Yuan et al., 2014). These findings support the potential utility of lidocaine’s anti-inflammatory properties in treating disorders involving excessive microglial activation, including neuropathic pain and neurodegenerative diseases.

Levobupivacaine also significantly modulates microglial activity. Studies demonstrate that it suppresses endotoxin-induced microglial activation and subsequent production of pro-inflammatory mediators (Huang et al., 2013). This action involves inhibition of the P2X7 receptor—a key regulator of microglial activation and cytokine release (Huang et al., 2015). The ability of levobupivacaine to suppress microglial activation suggests therapeutic potential in neuroinflammatory disorders such as chronic pain and neurodegenerative diseases. Studies further indicate that its effects on microglial responses are dose-dependent: lower concentrations produce anti-inflammatory benefits, whereas higher concentrations lead to more variable and potentially adverse outcomes (Toda et al., 2011). Whereas lidocaine and levobupivacaine suppress pro-inflammatory activity, certain local anesthetics can have cytotoxic consequences. Tetracaine promotes microglial apoptosis at specific concentrations, an effect that may contribute to neurotoxicity in certain pathological contexts (Matsuura et al., 2012).

Local anesthetics exert microglial modulation through overlapping yet distinct pathways. Lidocaine and levobupivacaine both suppress pro-inflammatory responses, but via different molecular targets: lidocaine primarily inhibits sodium channels and p38 MAPK, whereas levobupivacaine acts through P2X7 receptor antagonism. This mechanistic divergence may influence their efficacy in different neuroinflammatory settings. Tetracaine, however, induces apoptosis, revealing that structural variations among local anesthetics can shift outcomes from anti-inflammatory to cytotoxic. These findings argue for a mechanism-based approach to selecting local anesthetics in conditions where microglial activation is a key pathophysiological component.

### 2.3 Effects on oligodendrocytes

Local anesthetics exert complex effects on oligodendrocytes, influencing both myelin integrity and viability. Certain agents demonstrate protective properties by supporting myelin maintenance under pathological conditions; conversely, others induce cytotoxicity and apoptosis, particularly at elevated concentrations. These divergent actions underscore the necessity of agent-specific evaluation when considering anesthetic effects on oligodendrocyte function and their implications for therapeutic strategies.

Lidocaine exerts multifaceted effects on oligodendrocytes, influencing their survival and function, particularly during neuroinflammation and neuropathic pain. For example, by inhibiting pro-inflammatory cytokine production in microglia, lidocaine may indirectly protect oligodendrocytes from inflammation-mediated damage (Yuan et al., 2014). This protective mechanism may offer therapeutic benefit in conditions where oligodendrocytes are vulnerable to inflammatory injury. Nevertheless, lidocaine’s actions are not uniformly protective. The agent has demonstrated neurotoxic potential at elevated concentrations, an effect that may also compromise oligodendrocyte viability, though the underlying pathways require further investigation (Werdehausen et al., 2012).

Bupivacaine also influences oligodendrocyte physiology, though often indirectly. Studies show it impairs mitochondrial function in astrocytes—an effect that may extend to oligodendrocytes due to their functional interdependence in CNS homeostasis (Xing et al., 2018). Bupivacaine also reduces glycine transporter 1 (GlyT1) expression in spinal astrocytes—a change that may subsequently disrupt oligodendrocyte function by altering their extracellular microenvironment (Lu et al., 2022). While bupivacaine retains its therapeutic utility as a local anesthetic, these findings indicate it may carry adverse implications for oligodendrocyte function, particularly following prolonged exposure or at high concentrations.

The dual nature of local anesthetics—exerting both protective and detrimental effects on oligodendrocytes—emphasizes the need to elucidate the underlying cellular and molecular mechanisms. Despite their established clinical utility in pain management, these anesthetics require careful consideration regarding their impact on oligodendrocytes and other glial populations, particularly in vulnerable groups such as patients with neurodegenerative disorders or those with developing nervous systems. Therefore, further investigation is warranted to clarify the specific contexts in which these agents preserve or disrupt oligodendrocyte function, with the aim of maximizing therapeutic efficacy while minimizing neurotoxicity (Table 1).

**TABLE 1:** Effects of local anesthetics on glial cell function.

Local anesthetic	Glial cell type	Effect	Mechanism	References
Lidocaine	Astrocytes	Induces autophagy, reduces pro-inflammatory cytokines	Activates AMPK and upregulates SOCS3, inhibiting inflammatory signaling and promoting autophagic clearance	Yuan and Fei (2021), Zhang et al. (2017), Zheng et al. (2019)
	Microglia	Attenuates activation, reduces pro-inflammatory cytokines	Blocks voltage-gated sodium channels, inhibiting p38 MAPK phosphorylation and NF-κB nuclear translocation, thereby suppressing cytokine production	Cheng et al. (2011), Gu et al. (2008), Jeong et al. (2012), Yuan et al. (2014)
	Oligodendrocytes	Indirect protection from inflammation-induced damage	Reduces microglial pro-inflammatory cytokines, mitigating inflammatory injury to oligodendrocytes	Yuan et al. (2014)
Bupivacaine	Astrocytes	Impairs mitochondrial function, potentiates glutamate-induced calcium signaling	Uncouples oxidative phosphorylation and promotes ROS production, leading to ATP depletion and impaired glutamate uptake, which exacerbates neuronal excitotoxicity	Xing et al. (2018), Lai et al. (2022), Beard et al. (2021)
Levobupivacaine	Microglia	Suppresses activation, reduces pro-inflammatory mediators	Inhibits P2X7 receptor, attenuating microglial activation and cytokine release	Huang et al. (2013), Huang et al. (2015), Toda et al. (2011)
Tetracaine	Microglia	Induces apoptosis	Inhibits voltage-gated proton channels, triggering apoptotic pathways	Matsuura et al. (2012)

## 3 General anesthetics and glial cell function

### 3.1 Effects on astrocytes

General anesthetics exert profound effects on the central nervous system. Astrocytes—key regulators of neuronal homeostasis—represent an important cellular target for these effects. Their impacts span from inducing cytotoxicity and impairing neuronal support to providing protection against oxidative stress, often accompanied by disruptions in fundamental homeostatic processes like calcium signaling.

Isoflurane promotes astrocytic cytotoxicity through TREK-1 activation, which induces cell swelling and subsequent apoptosis (Guo et al., 2017). Isoflurane also compromises the supportive function of astrocytes in neuronal development, likely through its disruption of actin cytoskeletal dynamics (Ryu et al., 2014). Furthermore, isoflurane exposure in astrocytes is associated with exacerbated amyloid pathology in Alzheimer’s disease models, indicating potential long-term impacts on astrocyte function and neurodegenerative processes (Perucho et al., 2010).

In stark contrast to the cytotoxic and supportive deficits induced by volatile anesthetics, the intravenous agent propofol demonstrates protective capacities against astrocytic oxidative injury. Propofol, a widely used intravenous anesthetic, also significantly affects astrocytes. For instance, research indicates that it can protect these cells from oxidative stress by mitigating peroxide-induced inhibition of glutamate transport—a key mechanism for maintaining normal excitatory neurotransmission (Sitar et al., 1999). Propofol further mitigates DNA damage and apoptosis in astrocytes through peroxynitrite scavenging, indicating its potential to protect these cells from oxidative injury (Acquaviva et al., 2004). Propofol’s effects on astrocytes are not uniformly protective; the anesthetic can disrupt intracellular calcium homeostasis, leading to impaired cellular signaling and function (Barhoumi et al., 2007). These findings illustrate the context-dependent duality of propofol’s actions on astrocytes, encompassing both neuroprotective benefits via antioxidant mechanisms and potential adverse consequences through homeostatic disruption.

Anesthetic effects on astrocytes also involve modulation of glial fibrillary acidic protein (GFAP) expression and glutamate-aspartate transporter (GLAST) activity. For example, sevoflurane suppresses GFAP and GLAST expression in astrocytes via the JAK/STAT signaling pathway—a mechanism essential for astrocyte-mediated support of neuronal function (Wang et al., 2016). This suppression may compromise astrocytic glutamate buffering capacity, promoting excitotoxicity and neuronal injury—particularly in the developing brain. Repeated sevoflurane exposure also upregulates astrocytic glutamate transporter 1 (GLT-1), further inhibiting neurogenesis and amplifying the anesthetic’s neurotoxic potential (Kong et al., 2023). These findings underscore the profound and frequently adverse consequences of general anesthetics on astrocyte function, carrying important implications for brain development and the pathogenesis of neurodegenerative disorders.

General anesthetics exhibit a striking dichotomy in astrocytic responses: propofol enhances antioxidant defenses and mitigates oxidative damage, whereas isoflurane and sevoflurane impair glutamate handling and promote cytotoxicity. This divergence likely stems from their distinct molecular targets—TREK-1 activation by isoflurane versus peroxynitrite scavenging by propofol. The suppression of GLAST and GLT-1 by volatile anesthetics is particularly concerning in developing or aging brains, where glutamate dysregulation can accelerate neurodegeneration. These observations stress that anesthetic neurotoxicity is not merely a neuronal phenomenon but is critically mediated through astrocytic dysfunction.

### 3.2 Effects on microglia

General anesthetics exhibit divergent impacts on microglia, primarily polarizing between pro-inflammatory activation and anti-inflammatory or protective modulation.

Isoflurane induces microglial activation and upregulates pro-inflammatory cytokines including interleukin-6 via the nuclear factor-kappa B (NF-κB) pathway—a cascade that may amplify neuroinflammation and promote cognitive impairment (Zhang et al., 2013). Isoflurane activates microglia primarily through NF-κB pathway induction. This process begins with increased intracellular calcium (Jung et al., 2025), leading to IκB kinase activation and subsequent IκB degradation. Freed NF-κB translocates to the nucleus and promotes transcription of pro-inflammatory genes, including IL-6 and TNF-α. In developing brains, isoflurane also activates the NLRP3 inflammasome, leading to caspase-1-mediated IL-1β maturation (Wang et al., 2018). This cascade not only amplifies neuroinflammation but also disrupts synaptic pruning and neurogenesis, linking acute anesthetic exposure to long-term cognitive deficits. In neonatal models, isoflurane exposure potentiates microglial activation, resulting in impaired neurodevelopment and persistent cognitive deficits—indicating that exposure timing serves as a critical determinant of neurodevelopmental outcomes (Broad et al., 2016). Isoflurane triggers age-dependent activation of the NLRP3–caspase-1 pathway in microglia, reinforcing its involvement in neuroinflammatory processes and age-related cognitive decline (Wang et al., 2018).

Sevoflurane modulates microglial activity in a context-dependent fashion. Studies demonstrate that under conditions such as chronic intermittent hypoxia, sevoflurane downregulates hippocampal peroxisome proliferator-activated receptor gamma (PPAR-γ), thereby promoting microglia-driven neuroinflammation and cognitive impairment (Dong et al., 2018). Furthermore, sevoflurane itself can exhibit neuroprotective properties in different pathological settings, sevoflurane demonstrates neuroprotective properties in settings such as ischemic stroke, where it promotes microglial polarization toward an anti-inflammatory phenotype through the GSK-3β/Nrf2 pathway, thereby attenuating ischemic injury (Cai et al., 2021). These observations reveal the context-dependent duality of sevoflurane in regulating microglial responses, which may drive either neuroprotective or neuroinflammatory outcomes depending on the specific pathological environment.

Conversely, suppression of microglial activation or promotion of a protective phenotype is associated with other anesthetic contexts. Unlike volatile anesthetics, intravenous agents such as propofol demonstrate the ability to suppress microglial activation and mitigate neuroinflammatory signaling. This anti-inflammatory activity involves inhibition of NADPH oxidase, resulting in decreased reactive oxygen species (ROS) generation and attenuated microglial activation (Luo et al., 2013). Propofol also downregulates Toll-like receptor 4 (TLR4) and glycogen synthase kinase-3β (GSK-3β) signaling, thereby suppressing microglial activation and exerting neuroprotective effects in lipopolysaccharide (LPS)-induced neuroinflammation models (Gui et al., 2012). Nevertheless, propofol’s effects on microglia and neuroinflammation remain context-dependent, influenced by specific experimental parameters and the prevailing physiological or pathological state of the central nervous system.

### 3.3 Effects on oligodendrocytes

Oligodendrocytes, the myelinating cells of the central nervous system, play an essential role in neuronal signaling and function. Evidence indicates these cells exhibit marked vulnerability to general anesthetics, especially during developmental stages. In the developing brain, isoflurane triggers oligodendrocyte apoptosis, an effect linked to persistent deficits in myelination and overall neural function. Studies in neonatal rodent and primate models confirm that isoflurane exposure induces substantial oligodendrocyte loss, raising particular concern during sensitive periods of CNS maturation (Brambrink et al., 2012; Li et al., 2019). Isoflurane-induced oligodendrocyte apoptosis impairs myelin formation and may consequently lead to persistent cognitive and neurodevelopmental deficits (Creeley et al., 2014).

Furthermore, the vulnerability of oligodendrocytes to general anesthetics extends beyond isoflurane. Propofol, an intravenous anesthetic, also induces oligodendrocyte apoptosis in the developing brain. Studies in fetal and neonatal rhesus macaques demonstrate that propofol exposure triggers widespread oligodendrocyte cell death, comparable to isoflurane-induced effects (Creeley et al., 2013). These observations indicate that oligodendrocyte neurotoxicity may represent a shared property across multiple anesthetic classes. The associated impairment of oligodendrocyte development and myelination is particularly significant, given these cells' essential role in preserving neural circuit integrity and enabling efficient signal conduction throughout the brain.

Emerging evidence indicates that midazolam, a benzodiazepine sedative, disrupts oligodendrocyte development. Studies in larval zebrafish demonstrate that midazolam exposure impairs myelination by inhibiting oligodendrocyte maturation through the translocator protein (TSPO) pathway (Xu et al., 2022). Such myelination deficits may contribute to substantial neurodevelopmental impairments, particularly following early postnatal exposure. These observations collectively emphasize the susceptibility of oligodendrocytes to general anesthetics and underscore the importance of judicious anesthetic management—especially during developmental stages when disruptions to myelination can profoundly influence long-term cognitive function.

The vulnerability of oligodendrocytes to general anesthetics appears to be a class-wide phenomenon, observed with both volatile and intravenous agents. Isoflurane and propofol both induce apoptosis in developing oligodendrocytes, while midazolam disrupts maturation through TSPO modulation. This shared toxicity suggests that myelinating cells are particularly sensitive to anesthetic interference during critical developmental windows. The long-term functional consequences—impaired myelination and cognitive deficits—highlight the need for age-specific anesthetic protocols. Future work should distinguish between transient pharmacological effects and permanent developmental disruption (Table 2).

**TABLE 2:** Effects of general anesthetics on glial cell function.

General Anesthetic	Glial Cell Type	Effect	Mechanism	References
Isoflurane	Astrocytes	Induces cytotoxicity, increases amyloid pathology	Activates TREK-1 channels causing cell swelling and apoptosis; disrupts actin cytoskeleton, impairing neuronal support	Guo et al. (2017), Ryu et al. (2014), Perucho et al. (2010)
	Microglia	Activates microglia, increases pro-inflammatory cytokines	Activates NF-κB pathway via calcium-mediated IκB degradation and promotes NLRP3 inflammasome activation, driving cytokine production	Zhang et al. (2013), Wang et al. (2018), Broad et al. (2016)
	Oligodendrocytes	Induces apoptosis, disrupts myelination	Triggers apoptotic pathways during developmental windows, leading to impaired myelination	Brambrink et al. (2012), Li et al. (2019), Creeley et al. (2014)
Propofol	Astrocytes	Protects against oxidative stress, disrupts calcium homeostasis	Scavenges peroxynitrite and prevents peroxide-induced glutamate transport inhibition; may also alter calcium dynamics	Sitar et al. (1999), Acquaviva et al. (2004), Barhoumi et al. (2007)
	Microglia	Limits activation, attenuates neuroinflammatory responses	Inhibits NADPH oxidase and downregulates TLR4/GSK-3β signaling, reducing ROS and cytokine production	Luo et al. (2013), Gui et al. (2012)
Midazolam	Oligodendrocytes	Impedes development, impairs myelination	Acts via translocator protein (TSPO) pathway, disrupting oligodendrocyte maturation	Xu et al. (2022)

## 4 Contradictions and limitations

A critical appraisal of the literature necessitates an explicit acknowledgment of the significant contradictions and context-dependent outcomes that characterize this field. The effects of anesthetic agents on neuroinflammation are not intrinsic or uniform but are profoundly shaped by specific experimental and pathophysiological conditions. The divergent results reported for propofol and sevoflurane serve as paradigmatic examples. Propofol’s role can shift from anti-inflammatory to potentially pro-inflammatory or harmful primarily under conditions of supraclinical concentrations or prolonged exposure, which may induce cellular stress beyond compensatory mechanisms. Furthermore, its action is critically modified by the disease context; models of pre-existing metabolic dysfunction, advanced age, or sepsis may reveal adverse outcomes not observed in healthy systems, likely due to interactions with compromised cellular energetics or inflammatory priming. Similarly, the disparate findings regarding sevoflurane’s impact on microglia can be reconciled by examining key variables: concentration and duration (where low-dose preconditioning versus high-dose sustained exposure elicit opposite effects), the model system (acute injury versus chronic neurodegeneration models), and the baseline activation state of microglia at the time of exposure. These factors collectively determine whether sevoflurane modulates inflammation towards resolution or exacerbation. Beyond these specific agents, these principles form a unifying framework for interpreting contradictory data across other anesthetics. The field must therefore transition from binary classifications of “beneficial” or “detrimental” effects towards a conditional model. In this model, the net impact of any anesthetic is explicitly defined by the precise intersection of pharmacological parameters (dose, timing), biological context (specific disease state, comorbidities), and experimental system. Future research designed to systematically deconvolute these interactions is essential for developing predictable, tailored therapeutic approaches in perioperative neuromedicine.

## 5 Summary and perspectives

This review delineates the dual roles of local and general anesthetics on glial cells, underscoring that their neuroprotective or neurotoxic outcomes are not inherent but are governed by a complex interplay of agent-specific, temporal, and pathological factors. The evidence indicates that concentration, exposure timing, and the underlying cellular environment critically modulate glial responses, often flipping the balance between benefit and harm.

### 5.1 Key determinants of dual effects

The dichotomy in anesthetic action emerges from several contextual variables: 1) Concentration-Dependent Shifts: Low doses of lidocaine or levobupivacaine suppress neuroinflammation, whereas high concentrations of bupivacaine disrupt astrocytic mitochondria and promote excitotoxicity. Similarly, propofol exerts antioxidant effects at clinical doses but may perturb calcium homeostasis at elevated levels. 2) Developmental Vulnerability: The developing brain, especially oligodendrocytes, is highly susceptible to anesthetic-induced apoptosis and myelination deficits, as seen with isoflurane and propofol. Adult glia may display different thresholds and response patterns, urging age-specific anesthetic considerations. 3) Pathological Context: Preexisting neuroinflammation, metabolic stress, or neurodegeneration can dramatically alter glial responses. For instance, sevoflurane exacerbates inflammation in chronic hypoxia but promotes protective microglial polarization in ischemia via GSK-3β/Nrf2 signaling. 4) Cell-Type-Specific Mechanisms: Astrocytes, microglia, and oligodendrocytes engage distinct pathways—such as TREK-1 activation, NF-κB/NLRP3 inflammasome signaling, or TSPO modulation—highlighting the need to dissect glial subsets individually.

### 5.2 Remaining knowledge gaps

Despite advances, several questions remain: 1) Translational relevance of preclinical findings to human glial biology, especially in aged or comorbid populations. 2) Long-term consequences of transient anesthetic exposures on glial function and network homeostasis. 3) Effects of polypharmacy (combined anesthetics) on integrated glial responses. 4) Influence of sex, genetic background, and circadian rhythms on glial susceptibility. 5) Lack of real-time biomarkers to monitor perioperative glial activation states.

### 5.3 Future directions

To translate these insights into safer clinical practice, future research should: Systematically map concentration- and time-dependent glial responses across developmental stages and disease models. Develop human iPSC-derived glial-neuronal co-culture systems to better mimic human pathophysiology. Investigate pharmacological adjuncts (e.g., mitochondrial protectants, P2X7 or PPAR-γ modulators) to steer glial responses toward protection. Incorporate advanced *in vivo* imaging and omics approaches to decipher glial-specific networks affected by anesthetics. Conduct well-designed clinical studies correlating perioperative glial-related biomarkers with long-term neurological outcomes.

### 5.4 Clinical translation: from mechanisms to bedside decisions

The mechanistic landscape outlined in this review provides a framework for translating glial biology into clinically actionable strategies. The differential effects of anesthetics on astrocytes, microglia, and oligodendrocytes are not merely experimental observations but carry direct implications for perioperative care. First, agent selection can be tailored to patient-specific glial vulnerabilities. For example, in patients with pre-existing neuroinflammation (e.g., chronic pain, neurodegenerative disorders), lidocaine or propofol may be preferred due to their ability to suppress microglial activation and astrocytic inflammatory signaling. In contrast, high-dose or prolonged use of bupivacaine in elderly patients or those with metabolic syndrome might be approached with caution, given its potential to disrupt astrocytic mitochondrial function and promote excitotoxicity.

Second, developmental and pathological contexts dictate differential risk. The consistent evidence of oligodendrocyte apoptosis and myelination deficits induced by isoflurane and propofol in developing models reinforces the clinical principle of minimizing exposure duration and avoiding multiple agents in pediatric anesthesia. In adult patients with conditions characterized by glutamate dysregulation (e.g., stroke, traumatic brain injury) or demyelination, volatile anesthetics that suppress astrocytic glutamate transporters (e.g., sevoflurane) may warrant careful risk-benefit assessment.

Third, monitoring glial responses could inform personalized regimens. While still exploratory, the measurement of perioperative glial-derived biomarkers (e.g., GFAP for astrocytic injury, cytokines for microglial activation, or myelin-related proteins) may one day provide real-time feedback on glial state and guide anesthetic titration or adjunctive neuroprotective therapy.

Ultimately, moving from a neuron-centric to a glia-inclusive view of anesthetic pharmacology enables more nuanced clinical decision-making. By considering how anesthetics modulate glial autophagy, inflammation, metabolic support, and myelination, clinicians can better stratify patients based on their glial susceptibility and tailor anesthetic plans to preserve CNS homeostasis. This approach not only aims to reduce acute neurotoxicity but may also improve long-term neurological outcomes, particularly in vulnerable populations.

### 5.5 Conclusion

Glial cells are pivotal mediators of anesthetic effects in the CNS. Understanding the conditional nature of their responses—shaped by dose, timing, and context—is essential for designing safer anesthetic regimens. By integrating glial biology into anesthetic pharmacology, we can move toward personalized strategies that minimize neurotoxicity and harness endogenous protective mechanisms, ultimately improving perioperative neurological care and guiding future translational research.
